# Activation of UBEC2 by transcription factor MYBL2 affects DNA damage and promotes gastric cancer progression and cisplatin resistance

**DOI:** 10.1515/med-2023-0757

**Published:** 2023-10-11

**Authors:** Jiegen Long, Bin Zhu, Tao Tian, Linfei Ren, Yong Tao, Haitao Zhu, Dengwei Li, Yonghong Xu

**Affiliations:** Department of General Surgery, Affiliated Banan Hospital of Chongqing Medical University, Chongqing, 401320, China; Department of General Surgery, Affiliated Banan Hospital of Chongqing Medical University, No. 659, Yunan Road, Longzhouwan Street, Banan District, Chongqing, 401320, China

**Keywords:** MYBL2, UBE2C, gastric cancer, DNA damage, cisplatin resistance

## Abstract

Ubiquitin-conjugating enzyme E2 C (UBE2C) plays a carcinogenic role in gastric cancer (GC); yet, its role in cisplatin (DDP) resistance in GC is enigmatic. This study sought to probe into the impact of UBE2C on DDP resistance in GC and its concrete molecular mechanism in GC progression. Bioinformatics analysis was used to analyze differentially expressed mRNAs and predict upstream regulatory molecules in GC. Real-time quantitative reverse transcriptase polymerase chain reaction and western blot were used to detect the expression of UBE2C and MYB proto-oncogene like 2 (MYBL2). Dual luciferase and chromatin immunoprecipitation (ChIP) assays were used to verify the binding relationship. Cell counting kit-8 was used to detect cell viability and calculate IC_50_ values. Flow cytometry was used to detect the cell cycle. Comet assay was used to detect DNA damage. Western blot was used to detect the expression of DNA loss-related proteins (γ-H2AX, ATM/p-ATM). The knockdown of highly expressed UBE2C in GC cell lines could reduce cell viability, induce G2/M arrest, induce apoptosis, and promote DNA damage and DDP sensitivity. Bioinformatics analysis predicted that the substantially upregulated MYBL2 was an upstream transcription factor in UBE2C. The binding relationship between the UBE2C promoter region and MYBL2 was verified by dual luciferase and ChIP. Overexpression of UBE2C in the rescue experiment was found to reverse the inhibited GC progression and promoted DDP sensitivity brought by the knockdown of MYBL2. In conclusion, the MYBL2/UBE2C regulatory axis may be a potential way to overcome DDP resistance in GC.

## 1 Introduction

As the most common malignant neoplasm of the gastrointestinal tract, gastric cancer (GC) has the third highest mortality rate globally [1,2]. Cisplatin (DDP)-based chemotherapy is considered the first-line treatment for GC patients [3]; yet, as DDP resistance gradually increases after several sessions of treatment, cancer recurrence and metastases become inevitable [4]. In this case, since chemoresistance accounts for the reduced efficacy in the clinic [5,6], a full panorama of the molecular mechanisms of DDP resistance in GC is essential for survival improvement in GC patients. This study attempted to explore the mechanism affecting GC chemotherapy and provide some theoretical references that facilitate DDP treatment efficacy.

As one of the most effective broad-spectrum cancer therapeutics, DDP is widely used to treat ovarian, lung, cervical, head and neck, and other solid tumors [7]. Although some patients are sensitive to chemotherapy at the beginning, almost all patients with advanced GC develop resistance and relapse with continued use of chemotherapeutic agents [8]. The main target of DDP is DNA. As it enters tumor cells, DDP is hydrolyzed to diclodiaminoplatinum and then forms DDP-DNA adducts. When DNA is intra-/inter-linked through DNA-Pt-DNA structure, or DNA-protein is cross-linked through DNA-Pt-protein, the normal structure of DNA is damaged, which in turn inhibits DNA replication and transcription and induces apoptosis [9,10,11]. Thus, a better understanding of DDP-induced DNA damage could facilitate target identification, thereby improving the efficacy of DDP-based chemotherapy. Previously, some studies have explored at the cellular level how drug resistance in GC is regulated by mediating DNA damage. For instance, in GC, circAKT3 is discovered to inhibit DNA damage by targeting the miR-198/PIK3R1 axis, hence enhancing DDP resistance [4]. However, in GC, molecular mechanisms associated with DNA damage and DDP resistance have not been thoroughly investigated so far. Therefore, a deep exploration of the molecular mechanisms regulating DNA damage and chemoresistance is needed to improve the efficacy of chemotherapy in GC treatment.

Ubiquitin-conjugating enzyme E2 C (UBE2C) plays a vital role in tumorigenesis, such as regulating cell cycle progression and suppressing premature DNA replication [12]. Previous research manifested that UBE2C is highly expressed in pancreatic cancer [13], breast cancer [14], and esophageal squamous cell carcinoma [15]. Besides, Zhang et al. certified that UBE2C is overexpressed in GC tissues and associated with poor prognosis [16]. It has been reported that the expression of UBE2C is increased in DDP-resistant ovarian cancer cell lines. Silencing UBE2C can significantly reduce the resistance of DDP-resistant ovarian cancer cell lines to DDP and promote their apoptosis [17]. Although UBE2C is identified as a key regulator of tumor progression, the role in carcinogenesis and drug resistance of GC remains unclear.

MYB proto-oncogene like 2 (MYBL2) is a member of transcription factors, which plays a crucial role in the process of cell proliferation, differentiation, and cell cycle [18]. Meanwhile, MYBL2 overexpression has been observed in cancers such as bladder cancer [19] and prostate cancer [19], and it is currently used as a biomarker for poor prognosis in ovarian carcinoma [20]. However, it is still not clear about the mechanism of the MYBL2 gene in GC. In addition, it has been reported that the expression levels of UBE2C and MYBL2 show a strong positive correlation in a variety of cancers [21]. Therefore, we intended to explore the effect and mechanism of UBE2C on DDP resistance in GC. The aim of this study was to investigate the role of the MYBL2/UBE2C axis in DDP resistance in GC and its related regulatory mechanisms.

To verify the role of UBE2C in this setting, we performed cell molecular experiments and dissected the mechanism. The MYBL2/UBE2C axis discovered in this study was expected to provide different therapeutic approaches to improve chemoresistance in GC, hopefully emerging as a new target for GC treatment.

## 2 Materials and methods

### 2.1 Bioinformatics

With the mRNA expression data (normal: 32, tumor: 375) of GC downloaded from The Cancer Genome Atlas (TCGA) (https://portal.gdc.cancer.gov/), differential analysis was performed to obtain differentially expressed mRNAs (DEmRNAs) using the edgeR package (|log FC| > 2, false discovery rate (FDR) < 0.05). The target gene was confirmed by literature [12,22]. Subsequently, upstream transcription factors were predicted by hTFtarget (http://bioinfo.life.hust.edu.cn/hTFtarget#!/). The upstream targeted transcription factors were obtained by performing the intersection of upregulated DEmRNAs and predicted transcription factors and confirmed by Pearson correlation analysis, whose motif sites were predicted by JASPAR (http://jaspar.genereg.net/). According to the median value of UBE2C expression, the samples were divided into high- and low-expression groups. Gene set enrichment analysis (GSEA) was used to analyze the enrichment pathway of UBE2C high- and low-expression groups. The results were filtered by *P* value < 0.05 and FDR < 0.25.

### 2.2 Cell culture

Human normal gastric epithelial cell line GES-1 (immortalized cells were isolated and cultured from the gastric epithelial cells of newborn infants and transfected with SV40 virus) and human GC cell lines MKN45 (it is derived from the gastric lymph nodes of a 35-year-old woman with signet ring cell carcinoma. Human poorly differentiated GC cells highly express CD44 [23]) and HGC-27 (it is derived from undifferentiated GC tissues and can differentiate into functional endothelial cells under hypoxic conditions [24]) were purchased from Shanghai Cell Bank (China). SNU-1 (it is derived from poorly differentiated primary GC and is resistant to DDP [25]) and AGS (derived from untreated resected tumor fragments) cell lines were purchased from American Type Culture Collection (USA). All cells were maintained in Dulbecco’s modified eagle medium (HyClone, USA) containing 10% fetal bovine serum (Gibco, USA), which were cultured in a 37℃ incubator containing 5% CO_2_. The medium was changed every 48 h.

### 2.3 Cell transfection

pcDNA3.1-UBE2C (oe-UBE2C) and corresponding control (oe-NC) were synthesized by Shanghai Sangon Biotech (China). si-MYBL2 (siG000004605A-1-5) and corresponding negative control were purchased from RiboBio (China). si-MYBL2, oe-UBE2C, and corresponding negative controls were transfected into GC cells (HGC-27 and AGS) using Lipofectamine 2000 kit (Thermo Fisher Scientific, USA). 48 h later, the transfected cells were collected and prepared for the subsequent assays. The overexpression vector pcDNA3.1 used in this study was 5,428 bp in size, the promoter was CMV, and the vector resistance was Ampicillin. siRNAs are short and well-defined double-stranded RNA molecules that can be synthetically manufactured. The sequence of si-MYBL2 was as follows: sense: 5′-CCGUCCCUCCUACCAUAAATT-3′; antisense: 3′-UUUAUGGUAGGAGGGACGGTT-5′.

### 2.4 Real-time quantitative reverse transcriptase polymerase chain reaction (qRT-PCR)

Total RNA was extracted from GC cells using an RNA extraction kit (Vazyme, China), and the resulting RNA was reverse transcribed using a SuperScript IV Single Cell/Low-Input cDNA PreAmp kit (Thermo Fisher Scientific, USA). qRT-PCR analysis was performed in a StepOnePlus™ Real-time (ABI, USA) instrument using TB Green® Premix Ex Taq™ (Takara, Japan) with GAPDH as an internal reference. Primer sequences are available in Table 1. The experiments were performed for three times.

**Table 1:** Primer sequence for qRT-PCR

Gene	Sequence	
UBE2C	Forward primer	5′-GACCTGAGGTATAAGCTCTCGC-3′
	Reverse primer	5′-TTACCCTGGGTGTCCACGTT-3′
MYBL2	Forward primer	5′-CTTGAGCGAGTCCAAAGACTG-3′
	Reverse primer	5′-AGTTGGTCAGAAGACTTCCCT-3′
GAPDH	Forward primer	5′-TGACTTCAACAGCGACACCCA-3′
	Reverse primer	5′-CACCCTGTTGCTGTAGCCAAA-3′

### 2.5 Western blot

Western blot was conducted according to protocols mentioned in a previous study [26]. RIPA lysis buffer (Sigma, USA) plus protease inhibitors (Roche, Switzerland) were utilized to lyse cells. Bicinchoninic acid (Thermo Fisher Scientific, USA) was used for protein concentration quantification. The 40 μg extracted proteins were then separated by 10% sodium dodecyl sulfate-polyacrylamide gel electrophoresis (SDS-PAGE) and transferred to a polyvinylidene fluoride (PVDF) (Millipore, USA) membrane. After the membrane was blocked with 5% skim milk, it was maintained overnight with primary antibodies at 4℃, followed by incubating with secondary antibody the next day. Primary rabbit anti-human GAPDH (ab9485, 1:2,500), ATM/p-ATM (ab32420/ab81292, 1:2,000/1:50,000), γ-H2AX (ab81299, 1:5,000), UBE2C (ab252940, 1:1,000), and secondary goat anti-rabbit IgG (ab6721, 1:2,000) were purchased from Abcam (UK) and protein bands were visualized using the ECL Plus kit (Thermo Fisher Scientific, USA). ImageJ software was used to quantify the results. The experiments were carried out for three times.

### 2.6 Cell counting kit-8 (CCK-8)

Cells were cultured in 96-well plates (2 × 10^3^ cells/well) and 10 μL of CCK-8 reagent was added at 0, 24, 48, and 72 h, respectively. Absorbance was measured at 450 nm after 2 h of culture. For DDP resistance analysis, cells were treated with DDP (Adooq, USA) at various concentrations (0, 5, 10, 20, 40, and 80 μM) for 48 h. Phosphate-buffered saline (PBS) was utilized to dissolve DDP. Absorbance was measured and IC_50_ values were calculated [17]. The experiments were implemented for three times.

### 2.7 Flow cytometry

Cells investigated (5 × 10^5^ cells/well) were grown to 80% confluence, rinsed with cold PBS, and collected by scraping for flow cytometry.

Cell cycle: After pre-chilled PBS washes, cells were fixed with pre-chilled 70% ethanol for 2 h at 4℃. After 30 min of staining with propidium iodide (PI) solution, cells were then detected using a flow cytometer.

Apoptosis: Collected cells were stained with Annexin V-Fluorescein Isothiocyanate/PI according to the manuals (BD Pharmingen, USA). Detection was performed using a flow cytometer after 15 min. The assay was repeated for three times.

### 2.8 Comet assay

0.5% agarose was cooled to 40℃ and mixed with treated cells at a ratio of 10/1 (v/v) before spreading on slides. After that, cells were treated with lysis buffer for 1 h at 4℃ after cooling and then electrophoresed at 21 V for 30 min. Comets could then be observed under a fluorescence microscope after staining with PI, with comet and comet tail lengths being calculated using CaspLab software [27]. The experiments were repeated for three times.

### 2.9 Dual luciferase assay

Cells (3 × 10^4^ cells/well) were seeded into 24-well plates. pGL3-Basic-UBE2C-WT and pGL3-Basic-UBE2C-MUT luciferase reporter vectors (Promega, USA), si-NC, and si-MYBL2 were co-transfected into cells. Luciferase activity was measured after 48 h of culture. The experiment was repeated for three times.

### 2.10 Chromatin immunoprecipitation (ChIP)

According to the manufacturer’s instructions, ChIP experiments were performed using IP-grade anti-MYBL2 antibody (PA5-79713, 0.5 μg/mL, Invitrogen, USA) and corresponding Simple ChIP enzymatic chromatin IP kit (CST, USA). Specifically, formaldehyde was added to the cultured cells to fix the cells, and then, the cell samples were ultrasonically broken and collected. A part of the samples was incubated with IgG at 4℃ overnight, and a part of the samples was incubated with specific antibodies against MYBL2 at 4℃ overnight. The samples after incubation were precipitated with Protein Agar/Sepharose and centrifuged. After washing the impurities, the precipitate was decrosslinked at 65℃ overnight, and then, the DNA fragments were purified by phenol/chloroform extraction. Finally, PCR analysis was performed. Purified DNA was detected using ChIP-qPCR with primers shown in Table 2. The experiment was repeated for three times.

**Table 2:** Primer sequence for ChIP-qPCR

Primer	Sequence	
Site	Forward primer	5′-TGTGCCTGAGCGAGTTTGTA-3′
	Reverse primer	5′-TGGGTGAGGGTTATCTCGTCC-3′

### 2.11 Statistical analysis

Data were presented as mean ± standard deviation of three independent replicates (three independent biological replicates were performed for each set of experiments). Experimental data were processed using one-way analysis of variance or *T*-test using GraphPad 8.0 and *P*-values below 0.05 were interpreted as having a significant level. Tukey test was conducted as a post hoc test following one-way ANOVA.


**Ethics approval and consent to participate:** Our study did not require an ethical board approval because it did not contain human or animal trials.

## 3 Results

### 3.1 High expression of UBE2C in GC tissues and cells

**Figure 1:**
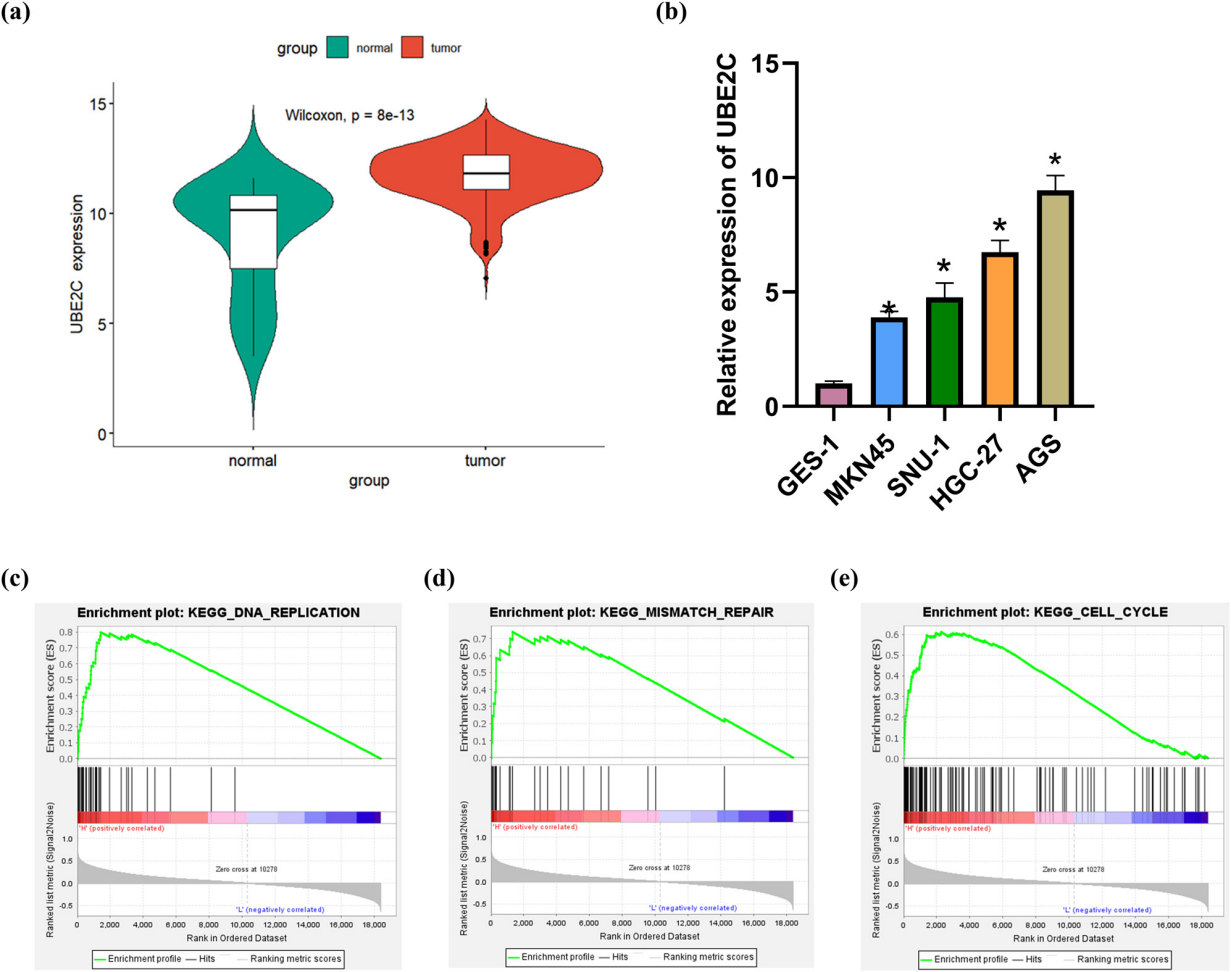
High expression of UBE2C in GC tissues and cells. (a) Expression of UBE2C in GC tissues and adjacent tissues in TCGA database (normal: 32, tumor: 375) (*P* = 8 × 10^−13^); (b) expression of UBE2C in human normal gastric epithelial cells (GES-1) and human GC cells (MKN45, SNU-1, HGC-27, and AGS) was detected by qRT-PCR for three times; (c–e) analysis of signaling pathways (DNA replication, mismatch repair, and cell cycle pathways) affected by UBE2C was conducted by using GSEA database; error bar: a graphical display of the variability of the data and is used to indicate the error or uncertainty of the measured value; the sample used as reference for relative comparison was GES-1 cells. *P < 0.05.

According to data retrieved from TCGA, UBE2C was upregulated in GC tissues (Figure 1a). Findings in previous studies also led us to conclude that UBE2C could promote the progression of GC [12,22]. Subsequent qRT-PCR results suggested that UBE2C expression was substantially higher in MKN45, SNU-1, HGC-27, and AGS cell lines than in the GES-1 cell line (Figure 1b). Since the most significant upregulation of UBE2C was found in HGC-27 and AGS, the following experiments were conducted on these two cell lines. As we analyzed the pathway involved in tumor development affected by UBE2C using GSEA, UBE2C turned out to be mainly enriched in DNA replication, mismatch repair, and cell cycle pathways. In this case, it was speculated that UBE2C promoted GC progression by affecting DNA damage (Figure 1c–e).

### 3.2 UBE2C promotes proliferation and DDP resistance of GC cells by regulating DNA damage

**Figure 2:**
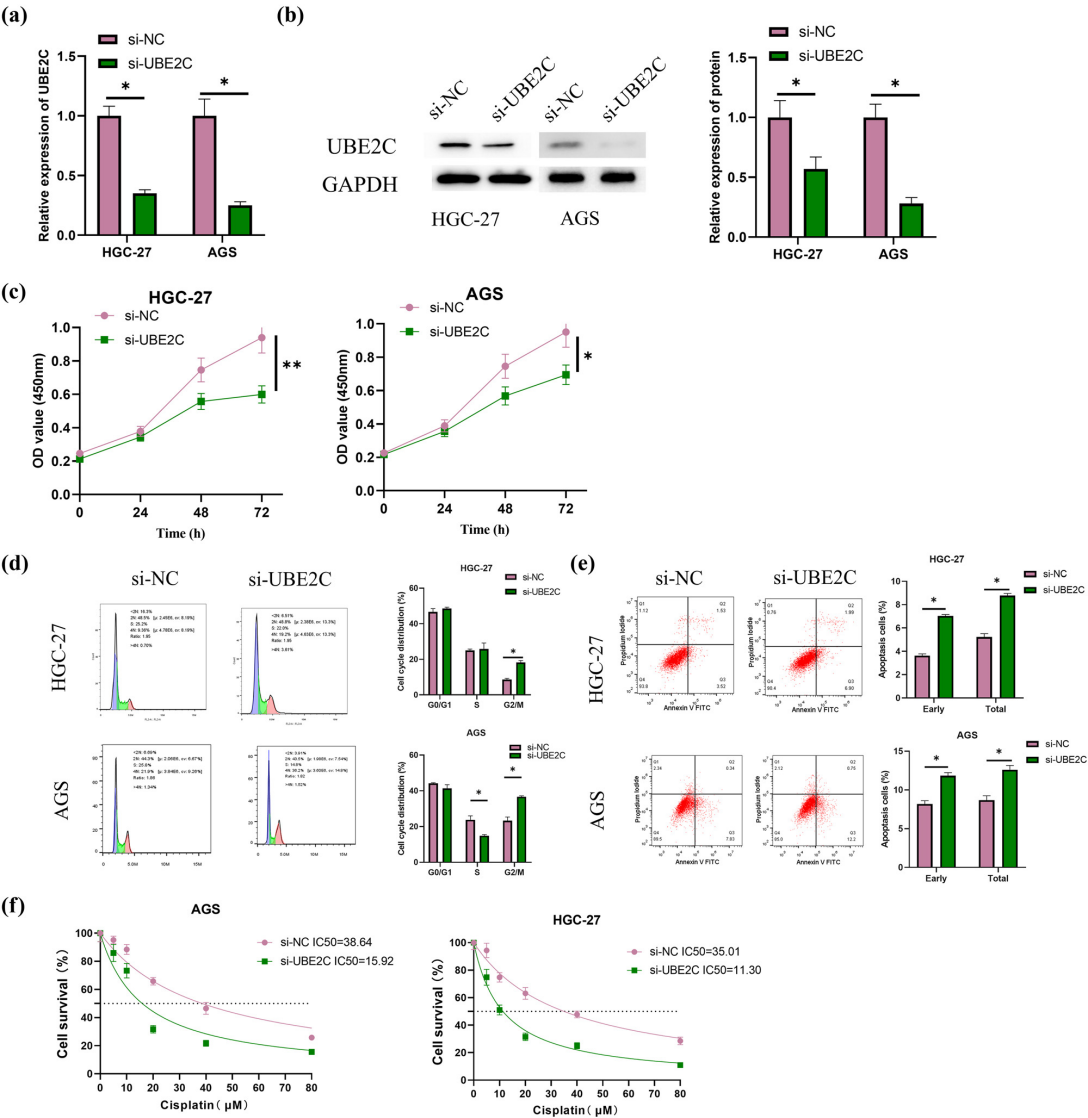

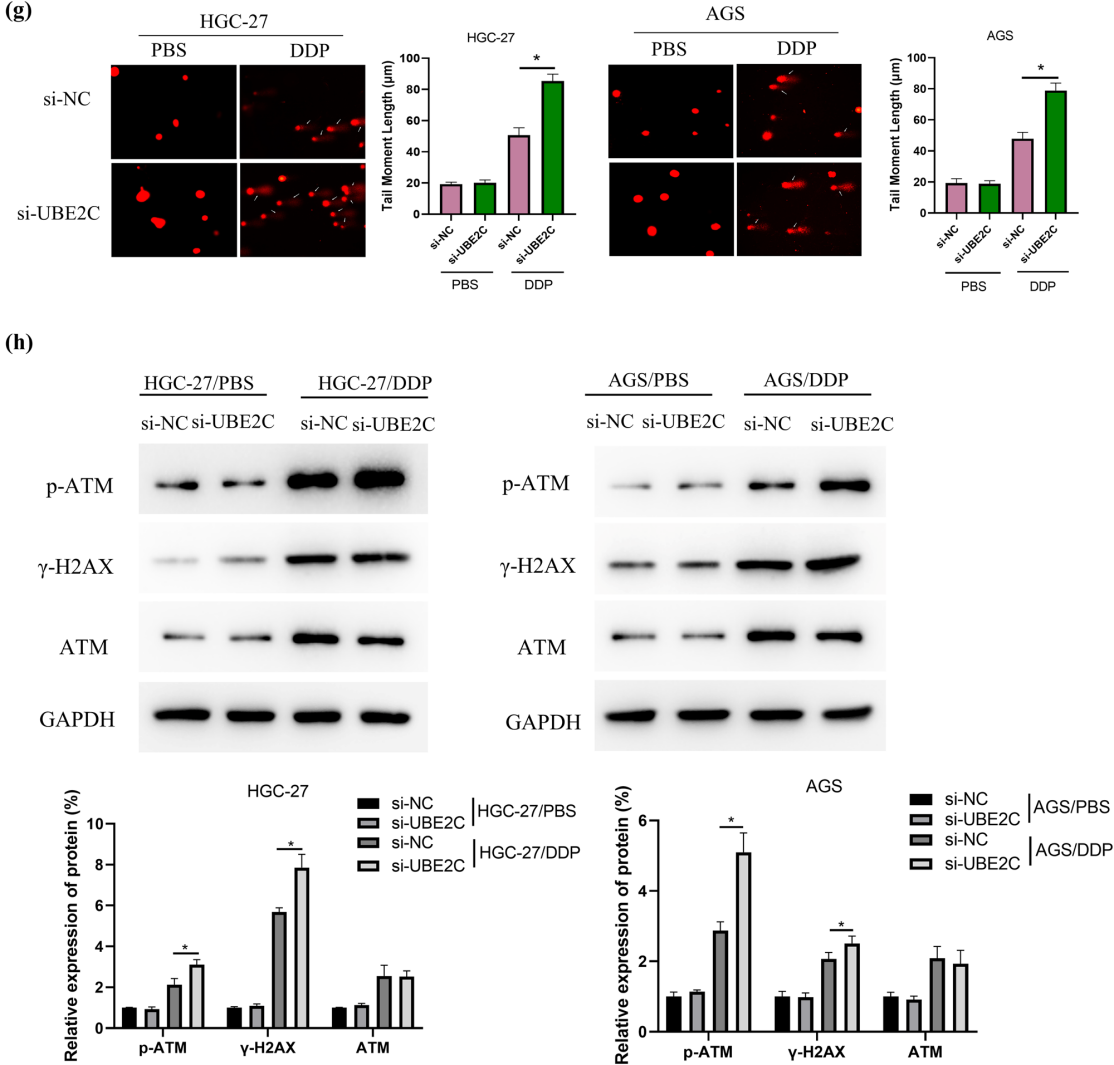
UBE2C promotes proliferation and DDP resistance in GC cells by regulating DNA damage. (a) Transfection efficiency of si-NC and si-UBE2C groups was examined via qRT-PCR for three times; (b) transfection efficiency of si-NC and si-UBE2C groups was examined via western blot for three times; (c) cell viability of HGC-27 and AGS cells was measured through CCK-8 for three times; (d and e) cell cycle distribution and apoptosis of si-NC and si-UBE2C groups in HGC-27 and AGS cells were examined by flow cytometry for three times; (f) IC_50_ value of si-NC and si-UBE2C groups in HGC-27 and AGS cells was detected by CCK-8; (g) DNA damage of si-NC and si-UBE2C groups in HGC-27 and AGS cells was measured by comet assay for three times (cells in five fields were counted); (h) expression of DNA damage-related proteins (ATM/p-ATM and γ-H2AX) was examined by western blot for three times; error bar: a graphical display of the variability of the data and is used to indicate the error or uncertainty of the measured value; the sample used as reference for relative comparison was si-NC group. For (g) and (h), cells were treated with DDP at various concentrations (0, 5, 10, 20, 40, 80 μM) for 48 h. *P < 0.05; **P < 0.01.

To explore the role of UBE2C in GC cells, si-NC and si-UBE2C were transfected into HGC-27 and AGS cells, respectively. After that, qRT-PCR and western blot were adopted to examine UBE2C expression, and a significant decrease in UBE2C was observed in HGC-27 and AGS cell lines transfected with si-UBE2C (Figure 2a and b). Besides, the viability of HGC-27 and AGS cells was found to decline after transfection with si-UBE2C according to the CCK-8 results (Figure 2c). Flow cytometry experiments also revealed that si-UBE2C transfection induced G2/M arrest in HGC-27 and AGS cells, and the AGS cells at S-phase were significantly reduced (Figure 2d). To ensure whether G2/M arrest resulting from UBE2C downregulation was associated with cell death, we further detected apoptosis and found that si-UBE2C transfection was able to promote apoptosis in HGC-27 and AGS (Figure 2e). Since a previous study reported that UBE2C could promote DDP resistance in ovarian cancer [17], cells were treated with different concentrations of DDP (0, 5, 10, 20, 40, and 80 μM) to investigate the effect of UBE2C on GC drug-resistance. According to the results, significantly reduced IC_50_ values were found in the HGC-27 and AGS cell lines transfected with si-UBE2C (Figure 2f). DNA damage is known to be strongly associated with DDP resistance [9], and we subsequently treated GC cells with DDP at a half-inhibitory concentration (17 μM) and detected DNA damage by comet assay. The results suggested that si-UBE2C was able to promote cellular DNA damage in HGC-27 and AGS cells treated with DDP (Figure 2g). The results of the western blot revealed that si-UBE2C transfection could promote the expression of two DNA damage-related proteins, namely γ-H2AX and p-ATM (Figure 2h). The above results prove that UBE2C promoted GC cell viability and DDP resistance by regulating DNA damage.

### 3.3 MYBL2 is a transcription factor upstream of UBE2C

**Figure 3:**
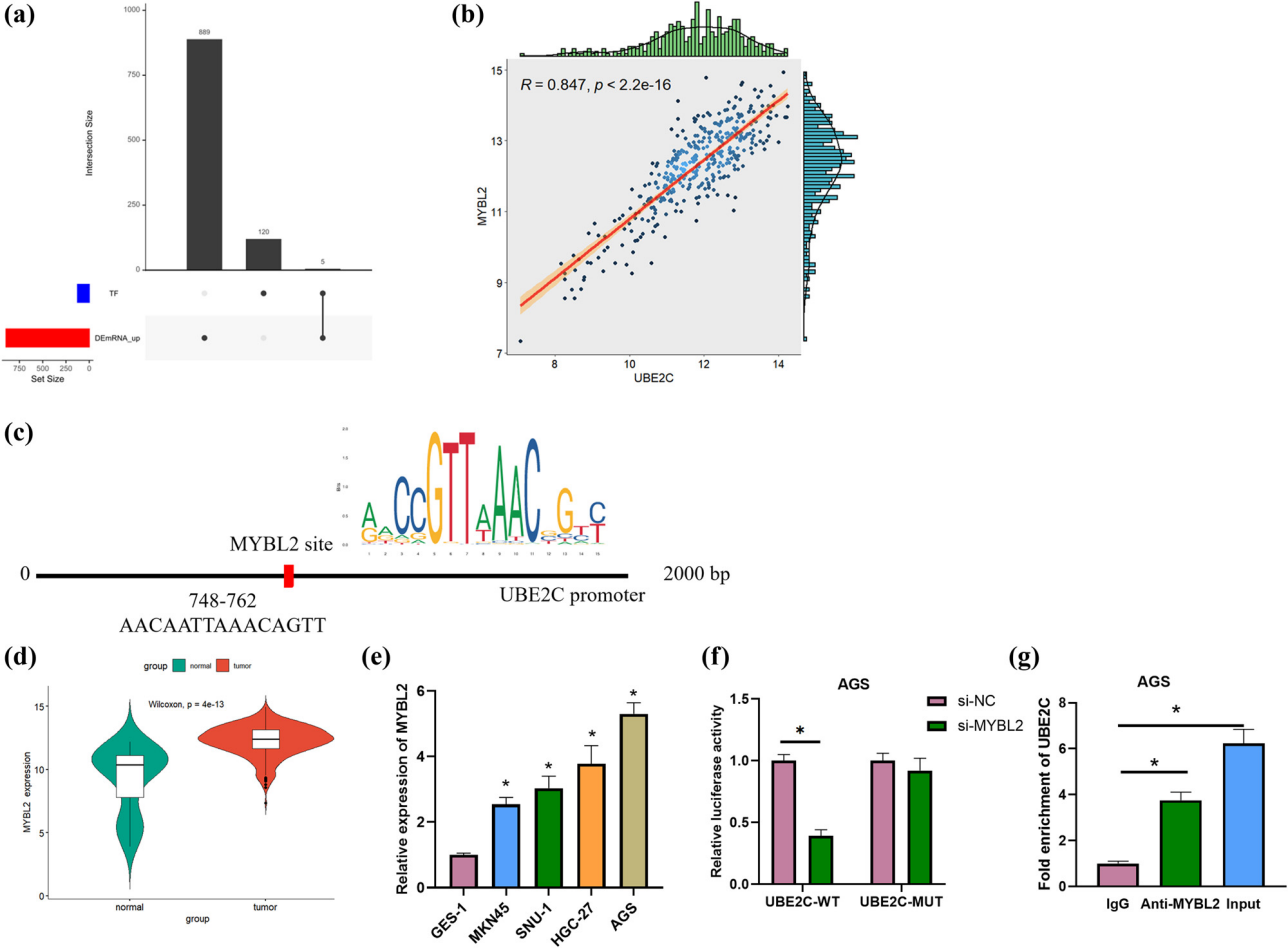
MYBL2 is a transcription factor upstream of UBE2C. (a) Upset plots of predicted upstream transcription factors of target genes and differentially expressed genes; the left bar chart (set size) represents the number of each set of genes, where DEmRNA_up represents differentially expressed up-regulated genes, and TF represents upstream transcription factors; the ordinate (intersection size) represents the number of intersections; points represent intersection; (b) Pearson correlation analysis was carried out on UBE2C and MYBL2 expression (*R* = 0.847, *P* < 2.2 × 10^−16^); The *R* value is positive, indicating that the two values are positively correlated, and the change trend is the same; the *P* value is the test value, *P* < 0.05, indicating a correlation; (c) targeting sites between UBE2C and MYBL2 were predicted by utilizing JASPAR; (d and e) MYBL2 expression in GC tissues and cells (MKN45, SNU-1, HGC-27, and AGS) was tested via bioinformatics and qRT-PCR, respectively; (f and g) binding relationship between UBE2C and MYBL2 in AGS cells was verified by dual luciferase and ChIP; error bar: a graphical display of the variability of the data and is used to indicate the error or uncertainty of the measured value; the sample used as reference for relative comparison in Figure 3e was GES-1 cells; the sample used as reference for relative comparison in Figure 3f was the si-NC group; the sample used as reference for relative comparison in Figure 3g was the IgG group. *P < 0.05.

For a comprehensive understanding of how UBE2C impacts GC, bioinformatics analysis was conducted to predict the upstream regulator of UBE2C. By intersecting with the upregulated genes in DEmRNAs, five potential transcription factors were finally obtained, including UBE2C (Figure 3a). It has been reported that the expression of UBE2C and MYBL2 was significantly positively correlated in a variety of cancers (including lung adenocarcinoma, lung squamous cell carcinoma, etc.) except GC [21]. Through analysis, we found that in GC, a positive correlation between UBE2C and MYBL2 expression was confirmed by correlation analysis (*R* = 0.847, *P* < 2.2 × 10^−16^) (Figure 3b). It is generally believed that the first 2,000 bp upstream of the gene is the promoter region of the gene. We predicted the binding site of MYBL2 in the UBE2C promoter region by JASPAR and finally selected the promoter binding sequence with a high comprehensive score (AACAATTAAACAGTT). The predicted sequence start site and end site were 748 and 762, respectively (Figure 3c). Bioinformatics analysis results and qRT-PCR assay results showed that MYBL2 expression was significantly upregulated in GC tissues and cells (Figure 3d and e). The phenotype of AGS was more pronounced in the above experiments, so AGS was next used for the experiment. Dual luciferase assay results revealed that si-MYBL2 was able to significantly reduce the luciferase activity of UBE2C-WT and had no significant effect on UBE2C-MUT (Figure 3f). ChIP assay results revealed that UBE2C enrichment was significantly improved with anti-MYBL2 compared with IgG (Figure 3g). The above results indicated that MYBL2 was a transcription factor upstream of UBE2C.

### 3.4 UBE2C activation by MYBL2 promotes GC and DDP resistance by regulating DNA damage

**Figure 4:**
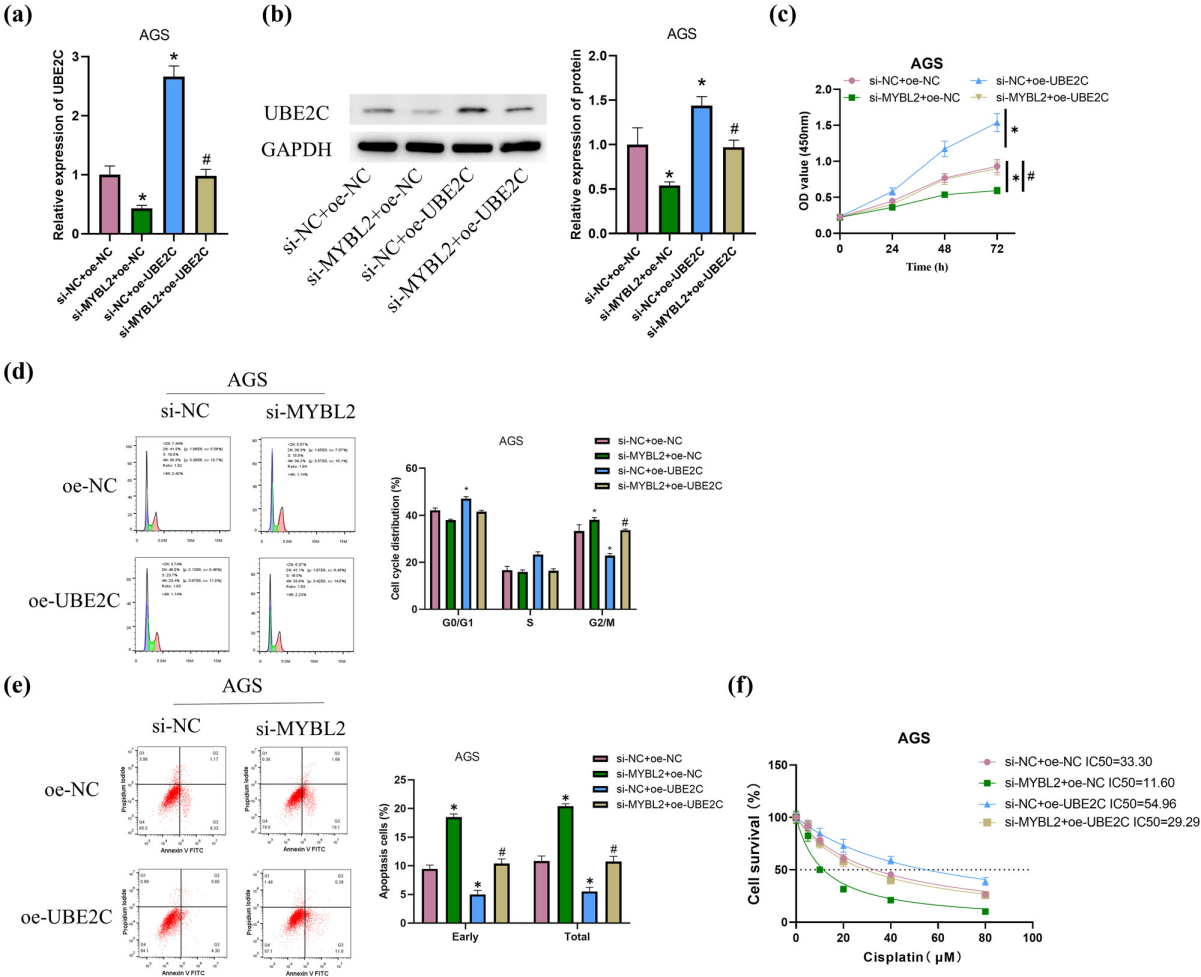

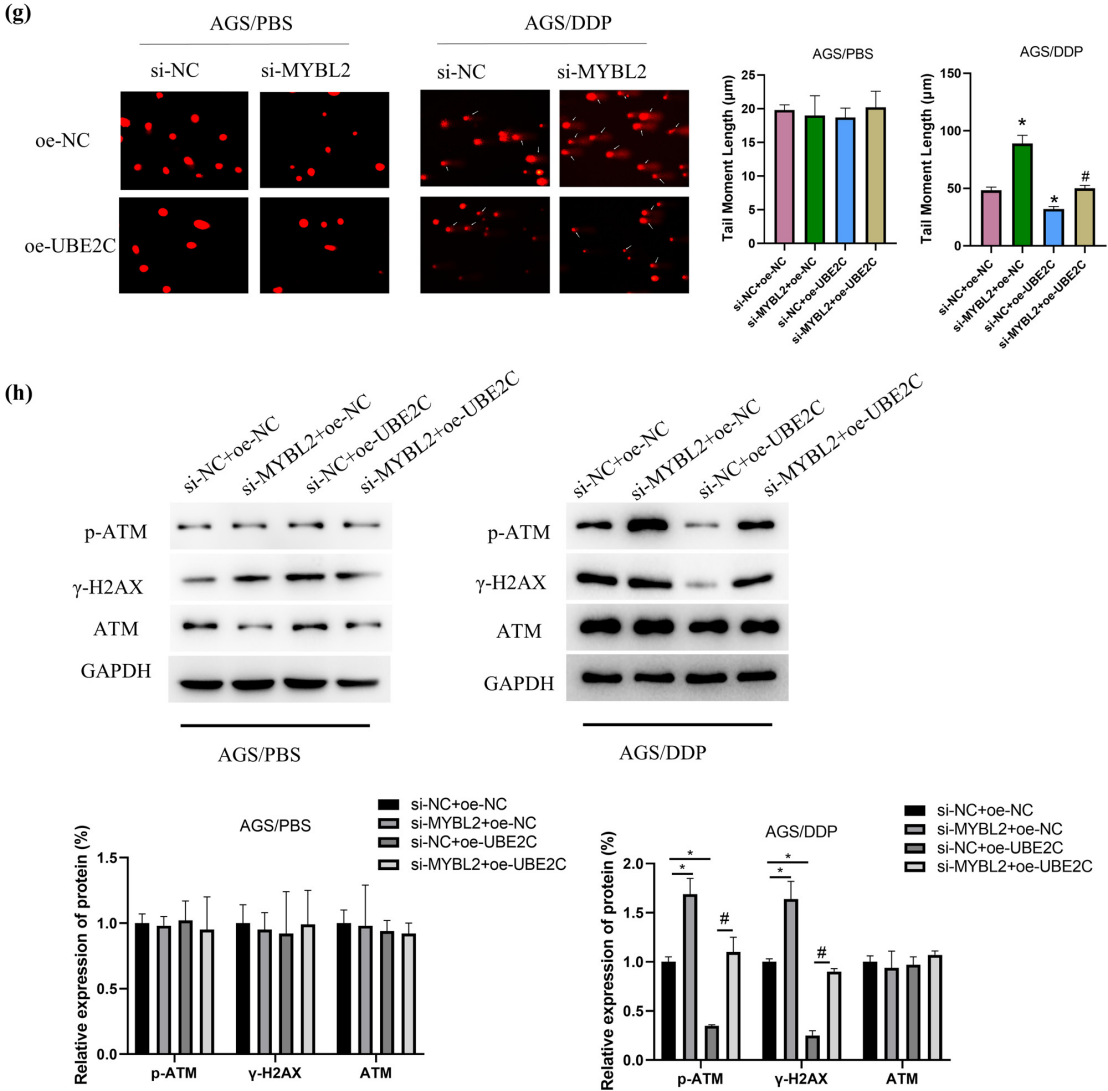
Activation of UBE2C by MYBL2 promotes GC progression and DDP resistance by regulating DNA damage. (a) Expression of UBE2C in each transfection group was detected by qRT-PCR for three times; (b) expression of UBE2C in each transfection group was detected by western blot for three times; (c) cell viability of the transfection groups was detected by CCK-8 for three times; (d and e) cell cycle distribution and apoptosis in each transfection group was detected by flow cytometry for three times; (f) IC_50_ values in the transfection groups were detected by CCK-8 for three times; (g) DNA damage was assessed in each transfection group by comet assay for three times (cells in five fields were counted); (h) DNA damage-associated proteins (ATM/p-ATM, γ-H2AX) were assessed by western blot; error bar: a graphical display of the variability of the data and is used to indicate the error or uncertainty of the measured value; For (f) and (g), cells were treated with DDP at various concentrations (0, 5, 10, 20, 40, 80 μM) for 48 h. *vs si-NC + oe-NC, ^#^vs si-MYBL2 + oe-NC; */# indicates *P* < 0.05.

To further investigate the effect of MYBL2 on GC progression through UBE2C, the following transfection groups were designed to treat AGS cells: si-NC + oe-NC/si-MYBL2 + oe-NC/si-NC + oe-UBE2C/si-MYBL2 + oe-UBE2C. qRT-PCR and western blot were used to detect the expression of UBE2C in each group, and the results revealed that UBE2C expression was inhibited in AGS cells transfected with si-MYBL2; yet, this reduced expression could be restored by oe-UBE2C transfection (Figure 4a and b). After adopting CCK-8 to assess cell viability, AGS cells transfected with si-MYBL2 saw a substantial decrease in cell viability, which could be restored by oe-UBE2C transfection (Figure 4c). Flow cytometry showed that the effect of si-MYBL2 on cell cycle and apoptosis, mainly promoting G2/M arrest and AGS apoptosis in HGC-27 and AGS cells, could be reversed by oe-UBE2C transfection (Figure 4d and e). To verify the effect of MYBL2 on drug resistance in GC cells, cells were subsequently treated with different concentrations of DDP (0, 5, 10, 20, 40, and 80 μM), and the results revealed that si-MYBL2 was able to significantly reduce the IC_50_ value of DDP in AGS cells, and the addition of oe-UBE2C could restore the IC_50_ value of cells to the si-NC + oe-NC group level (Figure 4f). Afterward, DNA damage was detected by comet assay. The results showed that si-MYBL2 could promote DNA damage in AGS cells, yet the promoting effect could be reversed by oe-UBE2C transfection (Figure 4g). According to the results of the western blot, which was used to detect the expression of DNA damage-associated proteins, namely γ-H2AX and p-ATM, si-MYBL2 transfection promoted their expression, while oe-UBE2C transfection could reverse this promoting effect (Figure 4h). The above results suggested that the MYBL2/UBE2C axis could promote DDP resistance in GC cells by regulating DNA damage.

## 4 Discussion

GC has a median survival rate of only 20%. UBE2C is the core ubiquitin-conjugating enzyme in the ubiquitin–proteasome system that promotes cell cycle progression. It is involved in regulating the degradation of proteins involved in cell cycle progression by interacting with anaphase-promoting complex/cyclosome [28,29,30]. Upregulation of UBE2C in breast cancer [31], non-small-cell lung cancer [30,32], and glioma [33] is discovered to promote the malignant progression of tumor cells. Previously, studies have investigated the effect of UBE2C on GC and found that the increased expression of UBE2C on GC can promote cell proliferation and inhibit apoptosis [34]. The conclusion reached in this study that UBE2C was highly expressed in GC tissues and cells was consistent with previous results. Previous studies have pointed out the link between chemosensitivity and UBE2C, where downregulation indicates better tumor cell chemosensitivity to chemotherapeutic agents [35,36]. In this study, we found that the knockdown UBE2C was able to inhibit cell viability and DDP resistance, promote cell apoptosis, and block cell cycle at the G2/M phase. Besides, comet assay and western blot assay revealed that silenced UBE2C in DDP-treatment cells could enhance DNA damage. Our observation could be part of the efforts in overcoming platinum resistance in GC by confirming the molecular mechanism of UBE2C in this setting.

To investigate the specific mechanism of UBE2C as an oncogene in GC progression, we predicted the upstream transcription factor of UBE2C by bioinformatics analysis and employed dual luciferase and ChIP assays to prove the binding relationship. As a key regulator of gene expression involved in tumorigenesis, MYBL2, a highly conserved member of the MYB transcription factors, plays a crucial role in mitotic progression, cell death, and proliferation [18,37,38]. For instance, knockdown of MYBL2 that is highly expressed in GC can inhibit cell proliferation and promote apoptosis [39]. Besides, MYBL2 is involved in tumor chemoresistance. For example, as an upstream transcription factor of CDCA8, MYBL2 promotes CDCA8 expression and hence improves the sensitivity of ovarian cancer cells to olaparib and DDP [40]. To sum up, our results indicated that MYBL2 was able to directly bind to the promoter region of UBE2C and activate transcription of UBE2C, thereby inhibiting cell apoptosis and DNA damage, promoting cell viability and DDP resistance in GC cells.

In summary, to our knowledge, this is the first study that investigated the effect of MYBL2/UBE2C regulatory axis on GC and found that MYBL2 could inhibit DNA damage and promote cell viability and DDP resistance in GC cells by activating UBE2C, which indicated that MYBL2/UBE2C axis was closely related to GC. However, this study has some limitations. First, it failed to further verify the reliability of the results at the animal and clinical levels. Second, the upstream regulatory molecules of MYBL2 and the relevant possible pathways involved have not been explored. Hopefully, this study could enhance the biological understanding of drug resistance in GC while providing evidence to support that MYBL2 and UBE2C are potential biomarkers and therapeutic targets for drug resistance in GC.

## References

[1] Kono Y, Kanzaki H, Iwamuro M, Kawano S, Kawahara Y, Okada H. Reality of gastric cancer in young patients: The importance and difficulty of the early diagnosis, prevention and treatment. Acta Med Okayama. 2020;74(6):461–6. 10.18926/AMO/61204 33361865

[2] Smyth EC, Nilsson M, Grabsch HI, van Grieken NC, Lordick F. Gastric cancer. Lancet. 2020;396(10251):635–48. 10.1016/S0140-6736(20)31288-5 32861308

[3] Shitara K. Chemotherapy for advanced gastric cancer: future perspective in Japan. Gastric Cancer. 2017;20(Suppl 1):102–10. 10.1007/s10120-016-0648-7 27699493

[4] Huang X, Li Z, Zhang Q, Wang W, Li B, Wang L, et al. Circular RNA AKT3 upregulates PIK3R1 to enhance cisplatin resistance in gastric cancer via miR-198 suppression. Mol Cancer. 2019;18(1):71. 10.1186/s12943-019-0969-3 PMC6441201 30927924

[5] Shah MA. Update on metastatic gastric and esophageal cancers. J Clin Oncol. 2015;33(16):1760–9. 10.1200/JCO.2014.60.1799 25918288

[6] Longley DB, Harkin DP, Johnston PG. 5-fluorouracil: mechanisms of action and clinical strategies. Nat Rev Cancer. 2003;3(5):330–8. 10.1038/nrc1074 12724731

[7] Kelland L. The resurgence of platinum-based cancer chemotherapy. Nat Rev Cancer. 2007;7(8):573–84. 10.1038/nrc2167 17625587

[8] Yu B, Gu D, Zhang X, Liu B, Xie J. The role of GLI2-ABCG2 signaling axis for 5Fu resistance in gastric cancer. J Genet Genomics. 2017;44(8):375–83. 10.1016/j.jgg.2017.04.008 PMC5604254 28847472

[9] Galluzzi L, Senovilla L, Vitale I, Michels J, Martins I, Kepp O, et al. Molecular mechanisms of cisplatin resistance. Oncogene. 2012;31(15):1869–83. 10.1038/onc.2011.384 21892204

[10] Martin LP, Hamilton TC, Schilder RJ. Platinum resistance: the role of DNA repair pathways. Clin Cancer Res. 2008;14(5):1291–5. 10.1158/1078-0432.CCR-07-2238 18316546

[11] Rabik CA, Dolan ME. Molecular mechanisms of resistance and toxicity associated with platinating agents. Cancer Treat Rev. 2007;33(1):9–23. 10.1016/j.ctrv.2006.09.006 PMC1855222 17084534

[12] Liu Y, Zhao R, Chi S, Zhang W, Xiao C, Zhou X, et al. UBE2C Is Upregulated by Estrogen and Promotes Epithelial-Mesenchymal Transition via p53 in Endometrial Cancer. Mol Cancer Res. 2020;18(2):204–15. 10.1158/1541-7786.MCR-19-0561 31662448

[13] Cao JZ, Nie G, Hu H, Zhang X, Ni CM, Huang ZP, et al. UBE2C promotes the progression of pancreatic cancer and glycolytic activity via EGFR stabilization-mediated PI3K-Akt pathway activation. J Gastrointest Oncol. 2022;13(3):1444–53. 10.21037/jgo-22-516 PMC9274053 35837197

[14] Qin T, Huang G, Chi L, Sui S, Song C, Li N, et al. Exceptionally high UBE2C expression is a unique phenomenon in basal-like type breast cancer and is regulated by BRCA1. Biomed Pharmacother. 2017;95:649–55. 10.1016/j.biopha.2017.08.095 28881292

[15] Palumbo A, Jr., Da Costa NM, De Martino M, Sepe R, Pellecchia S, de Sousa VP, et al. UBE2C is overexpressed in ESCC tissues and its abrogation attenuates the malignant phenotype of ESCC cell lines. Oncotarget. 2016;7(40):65876–87. 10.18632/oncotarget.11674 PMC5323199 27588470

[16] Zhang HQ, Zhao G, Ke B, Ma G, Liu GL, Liang H, et al. Overexpression of UBE2C correlates with poor prognosis in gastric cancer patients. Eur Rev Med Pharmacol Sci. 2018;22(6):1665–71. 10.26355/eurrev_201803_14578 29630110

[17] Li J, Zhi X, Shen X, Chen C, Yuan L, Dong X, et al. Depletion of UBE2C reduces ovarian cancer malignancy and reverses cisplatin resistance via downregulating CDK1. Biochem Biophys Res Commun. 2020;523(2):434–40. 10.1016/j.bbrc.2019.12.058 31875843

[18] Zhang X, Lv QL, Huang YT, Zhang LH, Zhou HH. Akt/FoxM1 signaling pathway-mediated upregulation of MYBL2 promotes progression of human glioma. J Exp Clin Cancer Res. 2017;36(1):105. 10.1186/s13046-017-0573-6 PMC5547476 28784180

[19] Li M, Liu Y, Liu J, Li W, Li N, Xue D, et al. Circ_0006332 promotes growth and progression of bladder cancer by modulating MYBL2 expression via miR-143. Aging (Albany NY). 2019;11(22):10626–43. 10.18632/aging.102481 PMC6914401 31756170

[20] Han J, Xie R, Yang Y, Chen D, Liu L, Wu J, et al. CENPA is one of the potential key genes associated with the proliferation and prognosis of ovarian cancer based on integrated bioinformatics analysis and regulated by MYBL2. Translational cancer research. 2021;10(9):4076–86. 10.21037/tcr-21-175 PMC8799161 35116705

[21] Dastsooz H, Cereda M, Donna D, Oliviero S. A Comprehensive Bioinformatics Analysis of UBE2C in Cancers. Int J Mol Sci. 2019;20(9):2228. 10.3390/ijms20092228 PMC6539744 31067633

[22] Jin Z, Zhao X, Cui L, Xu X, Zhao Y, Younai F, et al. UBE2C promotes the progression of head and neck squamous cell carcinoma. Biochem Biophys Res Commun. 2020;523(2):389–97. 10.1016/j.bbrc.2019.12.064 31870550

[23] Takaishi S, Okumura T, Tu S, Wang SS, Shibata W, Vigneshwaran R, et al. Identification of gastric cancer stem cells using the cell surface marker CD44. Stem Cells. 2009;27(5):1006–20. 10.1002/stem.30 PMC2746367 19415765

[24] Chen C, Hunag Z, Wang M, Huang Z, Chen X, Huang A, et al. Endothelial transdifferentiation of human HGC-27 gastric cancer cells in vitro. Oncol Lett. 2020;20(6):303. 10.3892/ol.2020.12166 PMC7573880 33093912

[25] Shrestha S, Song YW, Kim H, Lee DS, Cho SK. Sageone, a diterpene from Rosmarinus officinalis, synergizes with cisplatin cytotoxicity in SNU-1 human gastric cancer cells. Phytomedicine. 2016;23(13):1671–9. 10.1016/j.phymed.2016.09.008 27823632

[26] Tang T, Wang LX, Yang ML, Zhang RM. lncRNA TPTEP1 inhibits stemness and radioresistance of glioma through miR106a5pmediated P38 MAPK signaling. Mol Med Rep. 2020;22(6):4857–67. 10.3892/mmr.2020.11542 PMC7646932 33173989

[27] Sanches JGP, Song B, Zhang Q, Cui X, Yabasin IB, Ntim M, et al. The role of KDM2B and EZH2 in regulating the stemness in colorectal cancer through the PI3K/AKT Pathway. Front Oncol. 2021;11:637298. 10.3389/fonc.2021.637298 PMC8006351 33791221

[28] Alao JP. The regulation of cyclin D1 degradation: roles in cancer development and the potential for therapeutic invention. Mol Cancer. 2007;6:24. 10.1186/1476-4598-6-24 PMC1851974 17407548

[29] Lu Z, Hunter T. Ubiquitylation and proteasomal degradation of the p21(Cip1), p27(Kip1) and p57(Kip2) CDK inhibitors. Cell Cycle. 2010;9(12):2342–52. 10.4161/cc.9.12.11988 PMC3319752 20519948

[30] Xie C, Powell C, Yao M, Wu J, Dong Q. Ubiquitin-conjugating enzyme E2C: a potential cancer biomarker. Int J Biochem Cell Biol. 2014;47:113–7. 10.1016/j.biocel.2013.11.023 24361302

[31] Psyrri A, Kalogeras KT, Kronenwett R, Wirtz RM, Batistatou A, Bournakis E, et al. Prognostic significance of UBE2C mRNA expression in high-risk early breast cancer. A Hellenic Cooperative Oncology Group (HeCOG) Study. Ann Oncol. 2012;23(6):1422–7. 10.1093/annonc/mdr527 22056852

[32] Guo J, Wu Y, Du J, Yang L, Chen W, Gong K, et al. Deregulation of UBE2C-mediated autophagy repression aggravates NSCLC progression. Oncogenesis. 2018;7(6):49. 10.1038/s41389-018-0054-6 PMC6002383 29904125

[33] Jiang L, Bao Y, Luo C, Hu G, Huang C, Ding X, et al. Knockdown of ubiquitin-conjugating enzyme E2C/UbcH10 expression by RNA interference inhibits glioma cell proliferation and enhances cell apoptosis in vitro. J Cancer Res Clin Oncol. 2010;136(2):211–7. 10.1007/s00432-009-0651-z PMC11828233 19657671

[34] Zhang J, Liu X, Yu G, Liu L, Wang J, Chen X, et al. UBE2C is a potential biomarker of intestinal-type gastric cancer with chromosomal instability. Front Pharmacol. 2018;9:847. 10.3389/fphar.2018.00847 PMC6082955 30116193

[35] Wang C, Pan YH, Shan M, Xu M, Bao JL, Zhao LM. Knockdown of UbcH10 enhances the chemosensitivity of dual drug resistant breast cancer cells to epirubicin and docetaxel. Int J Mol Sci. 2015;16(3):4698–712. 10.3390/ijms16034698 PMC4394443 25739083

[36] Rawat A, Gopal G, Selvaluxmy G, Rajkumar T. Inhibition of ubiquitin conjugating enzyme UBE2C reduces proliferation and sensitizes breast cancer cells to radiation, doxorubicin, tamoxifen and letrozole. Cell Oncol (Dordr). 2013;36(6):459–67. 10.1007/s13402-013-0150-8 PMC13007477 24072565

[37] Xu C, He Z, Lin C, Shen Z. MiR-30b-5p inhibits proliferation and promotes apoptosis of medulloblastoma cells via targeting MYB proto-oncogene like 2 (MYBL2). J Investig Med. 2020;68(6):1179–85. 10.1136/jim-2020-001354 32690599

[38] Chen J, Chen X. MYBL2 Is targeted by miR-143-3p and regulates breast cancer cell proliferation and apoptosis. Oncol Res. 2018;26(6):913–22. 10.3727/096504017X15135941182107 PMC7844795 29268817

[39] Deng Q, Wu L, Li Y, Zou L. MYBL2 in synergy with CDC20 promotes the proliferation and inhibits apoptosis of gastric cancer cells. Adv Clin Exp Med. 2021;30(9):957–66. 10.17219/acem/135938 34358419

[40] Qi G, Zhang C, Ma H, Li Y, Peng J, Chen J, et al. CDCA8, targeted by MYBL2, promotes malignant progression and olaparib insensitivity in ovarian cancer. Am J Cancer Res. 2021;11(2):389–415. PMC7868764 33575078

